# Multimodal layer modelling reveals *in vivo* pathology in amyotrophic lateral sclerosis

**DOI:** 10.1093/brain/awad351

**Published:** 2023-10-10

**Authors:** Alicia Northall, Juliane Doehler, Miriam Weber, Igor Tellez, Susanne Petri, Johannes Prudlo, Stefan Vielhaber, Stefanie Schreiber, Esther Kuehn

**Affiliations:** Institute for Cognitive Neurology and Dementia Research (IKND), Otto-von-Guericke University Magdeburg, Magdeburg 39120, Germany; German Center for Neurodegenerative Diseases (DZNE), Magdeburg 39120, Germany; Institute for Cognitive Neurology and Dementia Research (IKND), Otto-von-Guericke University Magdeburg, Magdeburg 39120, Germany; German Center for Neurodegenerative Diseases (DZNE), Magdeburg 39120, Germany; Department of Neurology, Otto-von-Guericke University Magdeburg (OVGU), Magdeburg 39120, Germany; Institute for Cognitive Neurology and Dementia Research (IKND), Otto-von-Guericke University Magdeburg, Magdeburg 39120, Germany; Department of Neurology, Hannover Medical School (MHH), Hanover 30625, Germany; Department of Neurology, Rostock University Medical Centre, Rostock 18147, Germany; German Center for Neurodegenerative Diseases (DZNE), Rostock 18147, Germany; Department of Neurology, Otto-von-Guericke University Magdeburg (OVGU), Magdeburg 39120, Germany; German Center for Neurodegenerative Diseases (DZNE), Magdeburg 39120, Germany; Department of Neurology, Otto-von-Guericke University Magdeburg (OVGU), Magdeburg 39120, Germany; Center for Behavioral Brain Sciences (CBBS) Magdeburg, Magdeburg 39120, Germany; Institute for Cognitive Neurology and Dementia Research (IKND), Otto-von-Guericke University Magdeburg, Magdeburg 39120, Germany; Center for Behavioral Brain Sciences (CBBS) Magdeburg, Magdeburg 39120, Germany; German Center for Neurodegenerative Diseases (DZNE), Tübingen 72076, Germany; Hertie Institute for Clinical Brain Research (HIH), Tübingen 72076, Germany

**Keywords:** biomarker, neurodegeneration, individualised medicine, disease prediction, motor neuron disease

## Abstract

Amyotrophic lateral sclerosis (ALS) is a rapidly progressing neurodegenerative disease characterized by the loss of motor control. Current understanding of ALS pathology is largely based on post-mortem investigations at advanced disease stages. A systematic *in vivo* description of the microstructural changes that characterize early stage ALS, and their subsequent development, is so far lacking.

Recent advances in ultra-high field (7 T) MRI data modelling allow us to investigate cortical layers *in vivo*. Given the layer-specific and topographic signature of ALS pathology, we combined submillimetre structural 7 T MRI data (qT1, QSM), functional localizers of body parts (upper limb, lower limb, face) and layer modelling to systematically describe pathology in the primary motor cortex (M1), in 12 living ALS patients with reference to 12 matched controls. Longitudinal sampling was performed for a subset of patients. We calculated multimodal pathology maps for each layer (superficial layer, layer 5a, layer 5b, layer 6) of M1 to identify hot spots of demyelination, iron and calcium accumulation in different cortical fields.

We show preserved mean cortical thickness and layer architecture of M1, despite significantly increased iron in layer 6 and significantly increased calcium in layer 5a and superficial layer, in patients compared to controls. The behaviourally first-affected cortical field shows significantly increased iron in L6 compared to other fields, while calcium accumulation is atopographic and significantly increased in the low myelin borders between cortical fields compared to the fields themselves. A subset of patients with longitudinal data shows that the low myelin borders are particularly disrupted and that calcium hot spots, but to a lesser extent iron hot spots, precede demyelination. Finally, we highlight that a very slow progressing patient (Patient P4) shows a distinct pathology profile compared to the other patients.

Our data show that layer-specific markers of *in vivo* pathology can be identified in ALS patients with a single 7 T MRI measurement after first diagnosis, and that such data provide critical insights into the individual disease state. Our data highlight the non-topographic architecture of ALS disease spread and the role of calcium, rather than iron accumulation, in predicting future demyelination. We also highlight a potentially important role of low myelin borders, that are known to connect to multiple areas within the M1 architecture, in disease spread. Finally, the distinct pathology profile of a very-slow progressing patient (Patient P4) highlights a distinction between disease duration and progression.

Our findings demonstrate the importance of *in vivo* histology imaging for the diagnosis and prognosis of neurodegenerative diseases such as ALS.

## Teaser

ALS is a neurodegenerative disease characterized by motor control loss, typically resulting in death within 3 years. Current knowledge of ALS pathology is largely limited to the advanced disease stages accessible after death. Here, Northall *et al.* provide a systematic description of brain changes in living patients.

## Introduction

Amyotrophic lateral sclerosis (ALS) is a rapidly progressing neurodegenerative disease resulting in the loss of motor control, with a median survival time of 3 years.^1,2^ It affects both upper (UMN) and lower (LMN) motor neurons,^1^ and symptoms initially present focally in one body part. The disease spreads topographically, often first to the contralateral limb,^3^ eventually resulting in bulbar symptoms, which are associated with poorer outcomes.^4^ Current knowledge of ALS pathology is largely based on post-mortem evidence,^5-7^ therefore reflecting advanced disease stages. A detailed understanding of early stage ALS pathology is needed to understand disease mechanisms and to facilitate earlier diagnosis and prognosis. Such advances may identify novel therapeutic targets to slow disease progression.

Post-mortem studies have revealed abnormalities in the primary motor cortex (M1) in ALS, such as the depopulation of Betz cells in cortical layer 5(b),^5^ iron accumulation in deep cortex^6^ and increased intracellular calcium.^7^ As standard 1.5 T or 3 T MRI cannot detect these layer-specific features, our understanding of *in vivo* ALS pathology is limited and disease mechanisms are still debated, such as the focal hit versus multifocal pathology hypotheses.^8^ Recent advances in ultra-high field MRI at 7 T and above enable the automated assessment of anatomically-relevant cortical layers *in vivo*,^9-11^ allowing us to achieve *in vivo* histology. The present study aims to apply this approach to provide a systematic description of *in vivo* M1 pathology in early stage ALS.

Previous MRI studies have shown iron accumulation in M1 of ALS patients,^12-14^ which has been approximately localized to deep M1, where it reflects neuroinflammation and microglia activation,^6,15^ in addition to UMN impairment.^16^ Moreover, iron accumulation strongly affects the topographic area corresponding to the symptom onset site (i.e. first-affected).^17-19^ We previously showed that the topographic areas of M1 are microstructurally distinct in healthy adults and should therefore be considered cortical fields,^10^ which emphasizes the need for precise localization. Moreover, we showed that the lower-limb (LL), upper-limb (UL) and face (F) fields show distinct levels of age-related iron accumulation,^10^ highlighting the importance of using age-matched controls. In addition to iron, calcium is also dysregulated in ALS, where increased calcium is associated with glutamate excitotoxicity and motor neuron death in animal models.^20,21^ This excitotoxicity may reflect the increased excitability of human M1 in ALS.^22^ It is currently unclear how calcium dysregulation relates to iron accumulation and demyelination in ALS.

The low myelin borders that separate cortical fields in M1^23,24^ show interruptions in myelination at the depths where the Betz cells are located,^10,23^ which are known to be affected in ALS^5^ and are associated with different functional networks compared to cortical fields.^25^ As maladaptive proteins often propagate along highly myelinated neurons in neurodegeneration,^26^ these low myelin borders may act as natural boundaries to limit disease spread. Alternatively, the degeneration of these borders may contribute to topographic disease spread between cortical fields.^24^ An investigation into the stability of these low myelin borders in ALS is therefore of interest.

The present study aimed to provide a systematic description of *in vivo* pathology in ALS patients. We used topographic layer imaging^24^ to characterize pathology with respect to both cortical layers and fields.^10^ Submillimetre quantitative T1 (qT1) and quantitative susceptibility mapping (QSM) data, along with functional localizers, were collected from 12 ALS patients and 12 age-, gender-, education- and handedness-matched controls. We used increased positive QSM (pQSM) values as a validated marker of iron,^6,15^ increased qT1 values as a validated marker of (de)myelination^27^ and decreased (more negative) negative QSM (nQSM) as a marker of calcium.^28^ We extracted microstructural profiles from each cortical layer (superficial, layer 5a, layer 5b, layer 6) according to a previously published approach^9,10^ and calculated multimodal *in vivo* pathology maps. We then tested whether (i) the mean cortical thickness is decreased and/or the layer architecture of M1 is degenerated in patients compared to controls; (ii) pathological iron accumulation is layer-specific; (iii) pathological iron and calcium accumulation are topographic (i.e. largely restricted to the first-affected cortical field) or atopographic (i.e. not restricted to the first-affected cortical field); (iv) pathological iron and/or calcium accumulation hot spots precede demyelination; (v) low myelin borders between cortical fields are disrupted with disease progression, and whether they show high or low substance accumulation; and (vi) a very slow progressing patient shows a distinct multimodal pathology profile compared to other patients.

## Materials and methods

### Participants

Twelve ALS patients [six females, age: mean = 60.5, standard deviation (SD) = 12.7] and 12 healthy controls (six females, age: mean = 61.1, SD = 11.9) took part in the present study between June 2018 and December 2022. Controls were individually matched to patients based on age [±2 years; *t*(22) = −0.15, *P* = 0.883], handedness, gender and years of education [±4 years; patients: mean = 14.5, SD = 2.7; controls: mean = 15.4, SD = 2.7; *t*(22) = −0.82, *P* = 0.422]. On average, the revised ALS Functional Rating Scale (ALSFRS-R) score was 39.1 (SD = 6.5, range: 25–47) and patients were measured within 3 months of diagnosis (SD = 5, range: 0–14) and within 19 months of symptom onset (SD = 13, range: 2–48). Note that these group averages exclude one patient (Patient P4) with a very long disease duration (166 months between diagnosis and MRI). Participants underwent 7 T MRI and behavioural assessments. Three patients (Patients P1, P2 and P4) were measured again within 1 year (T2), while one patient (Patient P4) was also measured 8 months after T2 (T3). Patients were recruited from the University Clinic Magdeburg, Hannover Medical School and Rostock University Medical Center (Table 1). An experienced neurologist (S.V.) performed clinical assessments (Table 2). Of the 12 patients, seven had UL-onset (three right-lateralized, two left-lateralized, one bilateral), two had LL-onset (both left-lateralized) and three had bulbar-onset (B-onset; considered bilateral). Healthy controls were recruited from the DZNE database in Magdeburg and exclusion criteria included sensorimotor deficits, neurologic disorders and 7 T MRI contraindications. All participants gave written informed consent and were paid. The study was approved by the local Ethics Committee of the Medical Faculty of the University of Magdeburg.

**Table 1:** Clinical and demographic information for ALS patients (*n* = 12)

Patient	Age (years)	Education (years)	Gender	Handedness	Onset type	Onset side	Phenotype	PUMNS	El-Escorial	MRI symptoms (months)	MRI diagnosis (months)
P1	50	13	F	R	UL	L	LMND	1	Possible	12	5
P2	66	18	M	R	UL	L	UMND	3	Probable	12	0
P3	60	12	M	L	UL	L	UMND	11	Definite	12	0
P4	48	13	M	R	UL	R	Classical^a^	2	Definite	189	166
P5	77	14	F	R	UL	R	LMND	0	Definite	29	14
P6	73	15	M	R	UL	B	Classical	9	Definite	14	5
P7	74	15	F	R	LL	L	UMND	22	Definite	17	0
P8	36	16	M	R	LL	L	Classical	15	Probable	28	4
P9	52	12	F	R	UL	R	UMND	16	Possible	48	0
P10	53	21	M	R	B	B	Classical	2	Definite	32	12
P11	64	13	F	R	B	B	LMND	1	Possible	7	0
P12	72	12	F	R	B	B	LMND	1	Definite	2	−5

The Penn Upper Motor Neuron Scale (PUMNS)^29^ score indicates clinical signs of upper motor neuron involvement, with higher scores indicating greater impairment (mean = 6.92, SD = 7.46, range: 0–22). The El-Escorial criteria indicates the degree of certainty of ALS diagnosis.^30^ ‘MRI symptoms’ indicates the time (in months) between the MRI and symptom onset, while ‘MRI diagnosis’ indicates the time (in months) between MRI and formal diagnosis. Note that a negative MRI diagnosis indicates that the patient was scanned prior to a formal diagnosis. F = female; M = male; R = right; L = left; UL = upper limb; LL = lower limb; B = bulbar; B = bilateral; LMND = lower motor neuron dominant; UMN = upper motor neuron dominant, classical = both upper and lower motor neuron impairment.

^a^Note that Patient P4 first presented with an UL-isolated LMND phenotype, before progressing to a classical phenotype after 3 years from symptom onset.

**Table 2:** Clinical assessment scores for ALS patients (*n* = 12)

Patient	ALSFRS-R total	ALSFRS-R fine motor	ALSFRS-R gross motor	ALSFRS-R bulbar	KC stage	CNS-LS	CNS-BFS	DPR
P1	44	9	11	12	2A	14	22	0.24
P2	41	9	11	9	2B	11	31	0.64
P3	40	7	10	12	2A	15	32	0.73
P4	37	6	7	12	2B	7	21	0.06
P5	35	2	9	12	2A	7	24	0.48
P6	34	2	8	12	2B	7	23	1.00
P7	25	6	3	7	3	30	53	1.77
P8	45	12	9	12	2A	7	22	0.12
P9	42	10	9	12	2B	19	22	0.13
P10	33	9	9	7	3	10	62	0.45
P11	47	12	12	11	2A	25	28	0.17
P12	46	12	12	10	2A	7	32	1.00

The ALSFRS-R^31^ indicates disease severity, where lower values indicate greater impairment, with subscores for fine, gross and bulbar motor function. The King’s College (KC) stage^32^ indicates the stage of disease progression based on the ALSFRS-R score,^33^ where stage 2A reflects the involvement of one body part and that a clinical diagnosis has taken place, while stage 2B and stage 3 reflect the subsequent involvement of second and third body parts, respectively. The CNS-LS^34^ indicates the frequency of pseudobulbar episodes, with higher scores indicating greater impairment. The CNS-BFS^35^ indicates bulbar dysfunction, with higher scores indicating greater impairment. ALSFRS-R = ALS Functional Rating Scale-Revised; CNS-LS = Center for Neurologic Study-Lability Scale; CNS-BFS = Center for Neurologic Study-Bulbar Function Scale; Disease progression rate = 48 − (ALSFRS-R total)/MRI symptoms.

### 7 T MRI data acquisition

Data were collected using a 7 T MRI scanner (MAGNETOM, Siemens Healthcare) equipped with a 32-Channel Nova Medical Head Coil, located in Magdeburg, Germany. We acquired whole-brain, 0.7 mm isotropic resolution MP2RAGE images^36^ (sagittal slices, repetition time = 4800 ms, echo time = 2.01 ms, field of view read = 224 mm, GRAPPA 2, flip angle = 5°/3°, inversion time TI1/TI2 = 900/2750 ms, bandwidth = 250 Hz/Px). We also acquired whole-brain, 0.5 mm isotropic resolution susceptibility-weighted (SWI) images in 10/12 patients (transversal slices, repetition time = 22 ms, echo time = 9 ms, field of view read = 192 mm, GRAPPA 2, flip angle = 10°, bandwidth = 160 Hz/Px), as two patients could not be scanned any longer due to fatigue and discomfort (note that the SWI sequence was measured last). We also acquired a whole-brain, 1.5 mm isotropic resolution functional image (81 slices, repetition time = 2000ms, echo time = 25 ms, field of view read = 212 mm, GRAPPA 2, interleaved acquisition) using an EPI gradient-echo blood oxygen level-dependent (BOLD) sequence. The functional imaging involved a blocked design paradigm with 12-s periods of body part movements (left/right foot, left/right hand, tongue) alternated with 15-s of rest (as described previously^10^). Before scanning, participants were trained in the movements and patients with difficulty were encouraged to conduct a reduced amount of movement precisely. Instructions inside the scanner (grey background, black colour) asked participants to prepare (e.g. ‘prepare right foot’) before performing the movement (e.g. ‘move right foot’). Each movement was repeated four times, resulting in a total of 20 trials. Participants and patients wore fingerless braces covering the hands and forearms to reduce large movements of the hands and to facilitate fine motor movements. The total scanning duration was ∼75 min.

### Image processing

#### Structural preprocessing

CBS Tools (v3.0.8)^37^ in MIPAV (v7.3.0)^38^ was used to process the structural data. Skull stripping and dura estimation were used to remove extra-cranial tissue, which was manually refined using ITK-SNAP (v3.8.0). The TOADS algorithm^39^ was used to segment the brain into different tissue types, before the CRUISE module was used to estimate tissue boundaries,^40^ resulting in level set images. The distance field module was used to calculate mean cortical thickness (i.e. across cortical depth). The volumetric layering module^41,42^ was used to divide the cortex into 21 depths, according to the equivolume approach. M1 masks were manually delineated using ITK-SNAP, as described previously.^10^

#### Quantitative susceptibility mapping

After data quality checks, the SWI data of four patients (and the four matched controls) were excluded due to severe motion and truncation artefacts, leaving 16 participants for the SWI analyses. QSMbox (v2.0) was used to reconstruct QSM from the SWI images.^43^ In line with previous studies,^12,44^ QSM values were not normalized. We divided the QSM data into pQSM and nQSM values (as previously described^43^).

### Defining cortical layers

**Figure 1:**
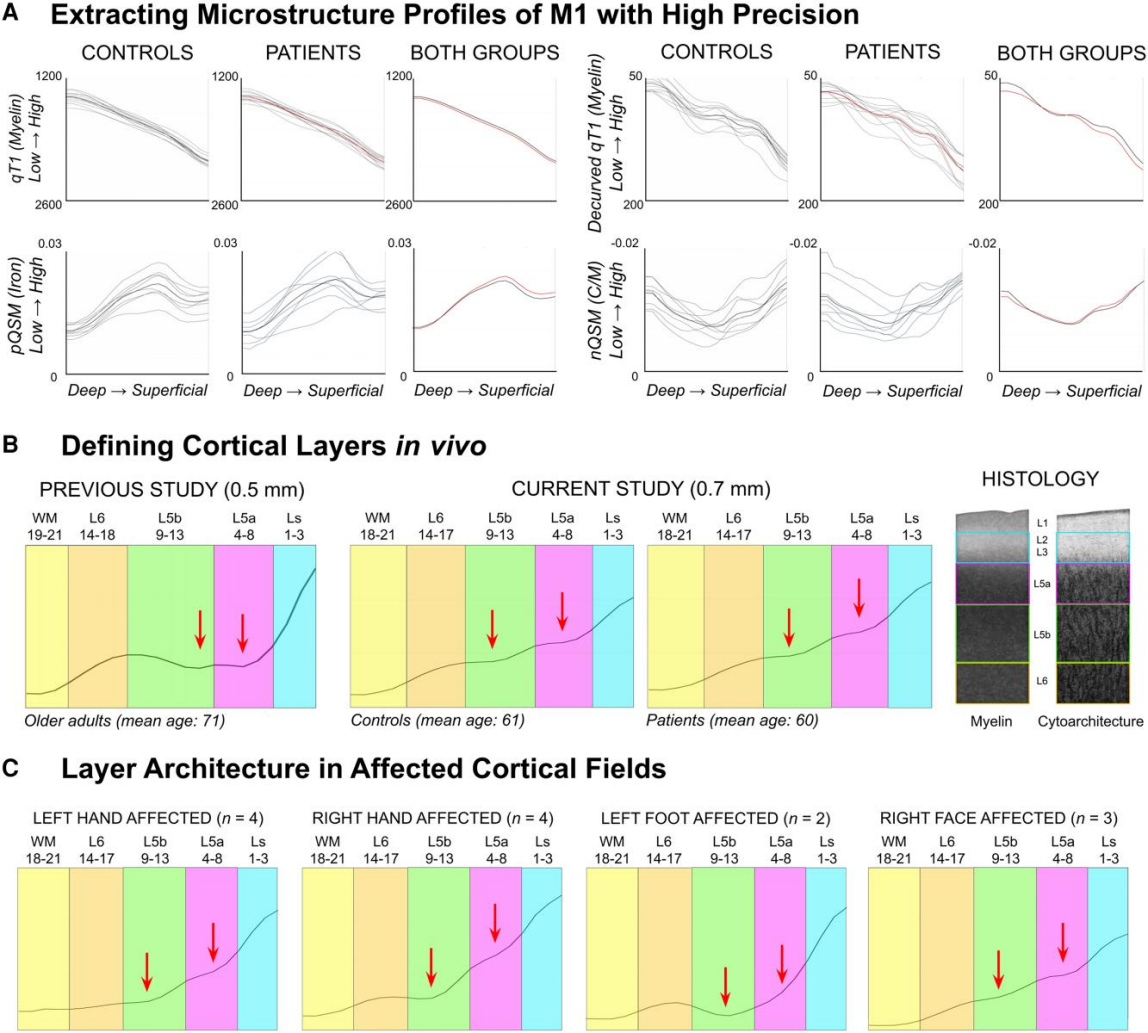
**Microstructure profiles of the left primary motor cortex (M1) in ALS patients and matched controls.** (**A**) ‘Raw qT1’ (i.e. not decurved; *n* = 12), decurved qT1 (*n* = 12), positive QSM (pQSM; *n* = 8) and negative QSM (nQSM; *n* = 8) data extracted across all cortical depths (*n* = 21) of left M1. The first and second columns show data for all controls and all patients, with the group mean plotted in bold black and red, respectively. The third column shows the mean group data of the controls and patients, with red lines representing the patients. Note that qT1 and pQSM are validated *in vivo* markers of myelin^27^ and iron^6,15^ content, respectively, while nQSM is largely considered to reflect calcium^28^ content. Also note that the profiles here represent the data of the entire M1, while in other figures and statistics the data used were averaged across cortical fields. (**B**) According to a previously published approach,^9^ we identified four compartments (‘layers’) based on the ‘decurved qT1’ profile: Ls = superficial layer including layers 2–3; L5a = layer 5a; L5b = layer 5b; L6 = layer 6. Note that Ls does not include layer 1 as it is inaccessible with MRI^9^ or layer 4, as it is absent in M1. We show the layer definitions for healthy controls (*n* = 12) and ALS patients (*n* = 12) in the present study (*centre*), as well as for older adults (*n* = 18) in our previous study^10^ (*left*). L5a and L5b were distinguished based on the presence of two small qT1 dips at the plateau of ‘decurved qT1’ values (indicating L5), while L6 was identified based on a sharp decrease in values before a further plateau indicating the presence of white matter. We show our layer approximations over schematic depictions (*right*) of M1 myelin^46^ and cell histological staining.^47^ (**C**) Despite differences in the shape of the profile in the affected fields compared to the average across fields in patients, layers can be similarly identified in the affected fields.

Using the simple curvature function in ParaView (v5.8.0), we calculated the mean curvature of the cortex. We then performed a vertex-wise linear regression analysis to predict qT1 values from mean curvature for each cortical layer in M1, on a subject-specific basis, according to an existing approach.^45^ The raw residuals of the regression models are referred to as ‘decurved’ qT1, following our previously published approach.^10^ We then applied a data-driven approach to identify cortical layers in M1 from group-averaged decurved qT1 profiles separately for patients and controls^9,10^ (see Fig. 1B for details and Supplementary Fig. 1 for right hemisphere).

### Functional data processing

The functional data were motion-corrected at acquisition using the Siemens ‘MoCo’ correction. Preprocessing was performed in Statistical Parametric Mapping 12 (SPM12), including smoothing [2 mm full-width at half-maximum (FWHM)] and slice-timing correction. Co-registration was performed using the automated registration tool in ITK-SNAP, with further manual refinement based on anatomical landmarks where necessary. A first-level analysis created t-statistic maps (t-maps) for each body part (e.g. left hand) based on contrast estimates (e.g. [1 0 0 0 0]). To create the subject-specific functional localizers of the cortical fields (e.g. left hand), we took the peak cluster of each t-map and removed overlapping voxels between localizers, as previously described.^10^ The functional localizers were then mapped onto the same subject-specific cortical surfaces as used for the structural data. We extracted layer-wise qT1 and signed QSM values from the cortical fields, resulting in microstructural profiles for each cortical layer (*n* = 4), each cortical field (*n* = 3) and each hemisphere (*n* = 2).

### Myelin border analysis

We identified myelin borders between the UL and F representations based on the highest qT1 value (i.e. lowest myelin) located between the peak t-values of the UL and F localizers, using a previous approach.^23,48^ For each cortical layer, we extracted qT1 and signed QSM values from the myelin borders and calculated the average qT1 and signed QSM values in the UL and F body part representations (UL + F/2), in order to compare values in the myelin border to the cortical fields.

### Layer-specific multimodal *in vivo* pathology mapping

**Figure 2:**
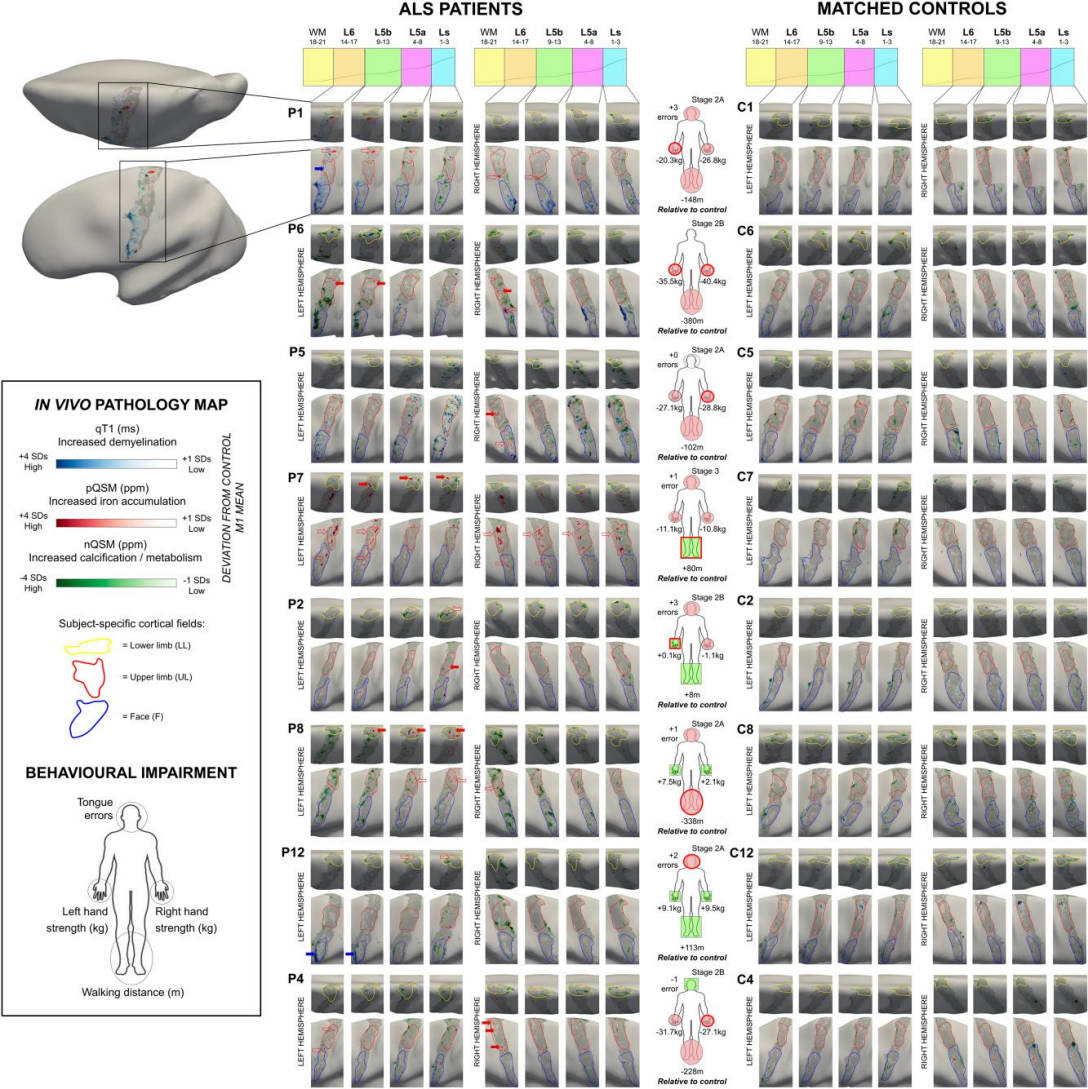
**Multimodal *in vivo* pathology maps in ALS patients.** Pathology maps were generated for each patient by thresholding the displayed value ranges at each layer to show increased qT1 and pQSM (+1 to +4 SD), and reduced nQSM (−1 to −4 SD), with respect to the mean M1 value of the matched control. Subject-specific cortical fields representing the lower limb, upper limb and face areas are outlined in yellow, red and blue, respectively. Pathology maps are shown for Patients P1, P2, P8, P12, P6, P7, P4 and P5 (*left*) and their corresponding matched controls C1, C2, C8, C12, C6, C7, C4 and C5 (*right*). Pathology maps: note that filled red and blue arrows indicate iron accumulation and demyelination in the first-affected cortical field, respectively. Unfilled red arrows indicate iron accumulation in cortical fields other than the first-affected field. Body maps: note that red-outlined circles on the body maps indicate the onset site (i.e. first-affected body part) of the patient. Filled red and green circles indicate impaired or better motor function in the circled body part compared to the matched control, respectively. The stage indicates the King’s College (KC) stage^32^ of disease progression based on ALSFRS-R score^33^: stage 2A reflects the involvement of one body part and that a clinical diagnosis has taken place, while stage 2B and stage 3 reflect the subsequent involvement of second and third body parts, respectively. L6 = layer 6; L5b = layer 5b; L5a = layer 5a; Ls = superficial layer; nQSM = negative QSM; pQSM = positive QSM; qT1 = quantitative T1.

Using topographic layer imaging, we created multimodal *in vivo* pathology maps on inflated cortical surfaces for each individual patient, in reference to the respective matched control (Fig. 2 and Supplementary Fig. 2). Pathology maps were calculated to identify disease hot spots indicated by demyelination (increased qT1, +1SD to +4 SDs), iron accumulation (increased pQSM, +1SD to +4 SDs) and calcium accumulation (more negative nQSM, −1 SD to −4 SDs) in each patient, relative to the mean value of the matched control. The reference values were layer-specific to control for microstructural differences between layers in M1.^10^ In addition, pathology maps are shown with individually localized cortical fields in M1, to account for microstructural differences between cortical fields.^10^

### Behavioural tests of motor function

All participants underwent body part-specific behavioural tests of motor function (as previously described^10^). To quantify LL function, the 6 Minute Walking Test (6MWT) was used to measure walking distance.^49^ To quantify UL function, a dynamometer^50^ was used to measure hand strength. In addition, the Purdue^51^ (Lafayette Instrument, model 32020A), Grooved^52^ (Lafayette Instrument, model 32025) and O’Connor^53^ (Lafayette Instrument, model 32021) pegboards were used to measure hand dexterity. To quantify bulbar (face, F) function, we used an automated tool that extracts features (e.g. errors) of lateral tongue movements from short video clips.^54^

### Statistical analyses

Statistical analyses were performed using IBM SPSS Statistics (v26, IBM, USA). Given the characteristic impaired motor function in ALS patients, we used one-tailed paired-samples *t*-tests to test for group differences in motor behaviour. Based on evidence of demyelination, iron and calcium accumulation in ALS,^5-7^ we used uncorrected one-tailed paired-samples *t*-tests to test for differences in cortical microstructure (i.e. qT1, QSM) between groups. Uncorrected *P*-values were used to avoid false-negatives due to the small sample size. We also report effect sizes with 95% confidence intervals. Note that paired-samples *t*-tests were used to account for dependencies in the data (i.e. matched patient-control pairs). We used two-tailed one-sample *t*-tests to test for differences in cortical microstructure between a single, very slowly progressing patient (Patient P4) and all other patients. We used linear regression models to test whether the layer-specific qT1, pQSM or nQSM predict whether a given cortical field is behaviourally affected. Individualized pathology maps were calculated by thresholding surfaces to show demyelination (increased qT1, +1 SD to +4 SDs), iron accumulation (increased pQSM, +1 SD to +4 SDs) and calcium accumulation (more negative nQSM, −1 SD to −4 SDs) in each patient relative to the mean M1 value of the matched control. Pathology estimations based on 1–4 SD are often used in clinical diagnostic imaging tools as they provide a detailed overview of how much the respective pathology differs from a control brain (e.g. AIRAmed Software, https://www.airamed.de/de/startseite).

## Results

### Impaired motor function in ALS patients

We quantified body part-specific motor function in 12 ALS patients and 12 matched controls (LL: 6MWT; UL: hand strength, pegboards; F: tongue kinematics). For three patients, follow-up behaviour measurements were performed. The results show that ALS patients are significantly impaired in motor function compared to matched controls (Supplementary Table 1).

### No significant M1 differences in patients when averaged across depths

We first tested whether the averaged qT1 and QSM values [averaged across cortical fields (LL, UL, F) and layers (L6, L5b, L5a, Ls) of M1] were significantly different in ALS patients compared to controls, to align with previous analyses.^12,16^ Using this approach, we show no significant differences in pQSM values between patients and controls in left M1 [patients: mean = 0.0172, SD = 0.0026; controls: mean = 0.0171, SD = 0.0028; *t*(7) = 0.2, *P* = 0.428, *d* = −0.037, 95% confidence interval (CI) (−1.02 0.94)] or right M1 [patients: mean = 0.0185, SD = 0.0027; controls: mean = 0.0172, SD = 0.0029; *t*(7) = 1.3, *P* = 0.114, *d* = −0.46, 95% CI (−1.46 0.53)]. There are also no significant differences in nQSM between patients and controls in left M1 [patients: mean = −0.0094, SD = 0.0019; controls: mean = −0.0085, SD = 0.0013; *t*(7) = 1.6, *P* = 0.081, *d* = 0.55, 95% CI (−0.45 1.55)] or right M1 [patients: mean = −0.0089, SD = 0.0021; controls: mean = −0.0087, SD = 0.0018; *t*(7) = 0.3, *P* = 0.375, *d* = 0.10, 95% CI (−0.88 1.08)]. There are also no significant differences in qT1 between patients and controls in left M1 [patients: mean = 1763.04, SD = 82.86; controls: mean = 1783.64, SD = 50.62; *t*(11) = 0.7, *P* = 0.764, *d* = 0.30, 95% CI (−0.54 1.14)] or right M1 [patients: mean = 1786.33, SD = 103.15; controls: mean = 1816.21, SD = 49.35; *t*(11) = 1.0, *P* = 0.834, *d* = 0.37, 95% CI (−0.47 1.21)]. Finally, there are no significant differences in mean cortical thickness between patients and controls, although there is a trend towards significantly reduced cortical thickness in the patients compared to controls in the F field (Supplementary Table 2).

### Layer-Specific M1 pathology in ALS patients

#### M1 layer architecture in ALS patients

As in our previous work,^10^ we used decurved qT1 values to identify four layers in M1 (Ls = superficial layer; L5a = layer 5a; L5b = layer 5b; L6 = layer 6) (Fig. 1B). Our data show that those four anatomically-relevant layers can be identified similarly for ALS patients and controls, where layers showed the same peaks (e.g. level set 11 for L5b) and relative thicknesses (e.g. level set 9–13 for L5b). In addition to the thickness, the principal layer architecture of M1 therefore also appears to be preserved in patients with early stage ALS (Fig. 1B), also in the affected cortical fields (Fig. 1C), although there are differences in the profile shape.

#### Pathological substance accumulation in M1 is layer-specific

**Figure 3:**
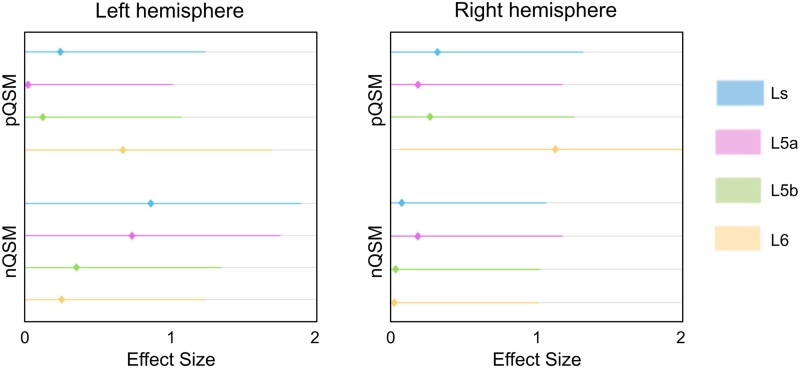
**Layer-specific differences in QSM between ALS patients (*n* = 8) and matched healthy controls (*n* = 8).** One-tailed paired-samples *t*-tests were used to investigate matched patient-control pair differences in microstructure (pQSM, nQSM, qT1) for each cortical layer (see Table 3 for statistics). Positive effect sizes indicate ‘more substance’ in patients compared to controls. Effect sizes (Cohen’s *d*) > 0.5 indicate medium effects, while effect sizes > 0.8 indicate large effects.^55^ L6 = layer 6; L5b = layer 5b; L5a = layer 5a; Ls = superficial layer; nQSM = negative QSM; pQSM = positive QSM; qT1 = quantitative T1.

We extracted qT1 and QSM values with high precision (Fig. 1A). Iron accumulation is a hallmark feature of ALS pathology^6,15^ and has been shown to particularly affect the deep region of M1. We show a significant increase in pQSM, specifically in L6 of left and right M1, in patients compared to matched controls [left M1: patients: mean = 0.0144, SD = 0.0020; controls: mean = 0.0128, SD = 0.0027; *t*(7) = 2.00, *P* = 0.043, *d* = 0.67, 95% CI (−0.33 1.68); right M1: patients: mean = 0.0165, SD = 0.0025; controls: mean = 0.0140, SD = 0.0019; *t*(7) = 2.00, *P* = 0.041, *d* = 1.13, 95% CI (0.07 2.18)]. These effects are moderate to large (Fig. 3). We show no significant differences or trends towards significance in pQSM between groups in the other layers (L5b, L5a and Ls) (see Table 3 for statistics).

**Table 3:** Layer-specific differences in M1 microstructure between patients (qT1: *n* = 12, QSM: *n* = 8) and matched healthy controls (qT1: *n* = 12, QSM: *n* = 8)

	ALS patients (*n* = 12)	Matched controls (*n* = 12)	Group difference			
	Mean (SD)	Mean (SD)	df	*t*	Sig.	*d* [95% CI]
pQSM						
Right L6	0.0165 (0.0025)	0.0140 (0.0019)	7	2.0	0.041^a^	1.13 [0.07 2.18]
Right L5b	0.0201 (0.0039)	0.0191 (0.0033)	7	0.8	0.220	0.28 [−0.71 1.26]
Right L5a	0.0195 (0.0041)	0.0187 (0.0039)	7	0.07	0.253	0.20 [−0.78 1.18]
Right Ls	0.0180 (0.0029)	0.0170 (0.0031)	7	1.0	0.170	0.33 [−0.65 1.32]
Left L6	0.0144 (0.0020)	0.0128 (0.0027)	7	2.0	0.043^a^	0.67 [−0.33 1.68]
Left L5b	0.0184 (0.0037)	0.0187 (0.0034)	7	−0.40	0.360	−0.13 [−1.07 0.90]
Left L5a	0.0191 (0.0030)	0.0190 (0.0037)	7	0.02	0.492	0.03 [−0.95 1.01]
Left Ls	0.0170 (0.0028)	0.0177 (0.0028)	7	−0.60	0.727	−0.25 [−1.23 0.73]
nQSM						
Right L6	−0.0075 (0.0034)	−0.0074 (0.0019)	7	0.20	0.441	0.04 [−0.94 1.02]
Right L5b	−0.0064 (0.0027)	−0.0063 (0.0015)	7	0.20	0.443	0.05 [−0.93 1.03]
Right L5a	−0.0088 (0.0021)	−0.0084 (0.0020)	7	0.50	0.303	0.20 [−0.79 1.18]
Right Ls	−0.0128 (0.0018)	−0.0126 (0.0025)	7	0.30	0.387	0.09 [−0.89 1.07]
Left L6	−0.0082 (0.0029)	−0.0076 (0.0016)	7	0.60	0.294	0.26 [−0.73 1.24]
Left L5b	−0.0069 (0.0028)	−0.0061 (0.0014)	7	0.90	0.210	0.36 [−0.63 1.35]
Left L5a	−0.0092 (0.0019)	−0.0080 (0.0013)	7	2.0	0.040^a^	0.74 [−0.28 1.75]
Left Ls	−0.0134 (0.0016)	−0.0121 (0.0014)	7	2.4	0.024^a^	0.87 [−0.16 1.89]
qT1						
Right L6	1526.11 (93.37)	1558.45 (57.37)	11	1.1	0.860	0.42 [−0.43 1.26]
Right L5b	1676.91 (95.40)	1708.91 (59.83)	11	1.4	0.903	0.40 [−0.44 1.25]
Right L5a	1863.87 (110.11)	1895.49 (52.47)	11	1.0	0.826	0.37 [−0.48 1.21]
Right Ls	2078.43 (132.64)	2101.98 (40.33)	11	0.6	0.706	0.24 [−0.60 1.08]
Left L6	1501.84 (69.33)	1517.70 (57.46)	11	0.6	0.728	0.25 [−0.59 1.09]
Left L5b	1647.98 (79.63)	1671.06 (56.68)	11	0.9	0.805	0.33 [−0.51 1.18]
Left L5a	1863.43 (54.68)	1841.15 (93.62)	11	0.7	0.753	−0.29 [−1.13 0.55]
Left Ls	2061.17 (113.51)	2082.36 (44.43)	11	0.6	0.707	0.25 [−0.59 1.09]

One-tailed paired-samples *t*-tests were used to investigate matched patient-control pair differences in microstructure (pQSM, nQSM, qT1) for each cortical layer. Note that effect sizes (Cohen’s *d*) > 0.5 indicate medium effects, while effect sizes > 0.8 indicate large effects.^55^ L6 = layer 6; L5b = layer 5b; L5a = layer 5a; Ls = superficial layer; CI = confidence interval; nQSM = negative QSM; pQSM = positive QSM; qT1 = quantitative T1; SD = standard deviation.

^a^Significance at the 0.05 uncorrected level, while no tests show significance at the Bonferroni-corrected level (0.05/32 = 0.002).

### Iron accumulates in first-affected cortical field

Using topographic layer imaging, we created *in vivo* pathology maps for each individual ALS patient in reference to their respective matched control (Fig. 2 and Supplementary Fig. 2 for patients with qT1 data only). These maps reveal that demyelination is minimal compared to iron and calcium accumulation. As expected,^17-19^ iron accumulation is higher in the first-affected cortical field compared to the other fields (excluding the contralateral field given evidence of highly symmetric pQSM increases^19^) (filled red arrows in Fig. 2). This effect is specific to L6 [first-affected: mean = 8.95, SD = 7.94, others: mean = 2.49, *SD* = 1.68; *t*(6)= 2.81, *P* = 0.013, *d* = 0.95, 95% CI (−0.04 2.22)], while there are no significant differences in L5b [first-affected: mean = 6.66, SD = 8.19, others: mean = 2.78, SD = 2.60; *t*(6)= 1.82, *P* = 0.056, *d* = 0.54, 95% CI (−0.27 1.50)], L5a [first-affected: mean = 4.66, SD = 3.56, others: mean = 3.54, SD = 2.45; *t*(6)= 1.50, *P* = 0.089, *d* = 0.31, 95% CI (−0.25 0.95)] or Ls [first-affected: mean = 3.11, SD = 2.44, others: mean = 3.02, SD = 1.94; *t*(6) = 0.17, *P* = 0.436, *d* = 0.03, 95% CI (−0.57 0.65)]. However, based on visual inspection, iron accumulation also occurs in unaffected body parts (non-filled red arrows in Fig. 2). An exception is Patient P12, who does not show iron accumulation, but shows demyelination in the F field, which may correspond to the bulbar-onset type. Linear regression analyses show that pQSM pathology (nor qT1 pathology or nQSM pathology) does not significantly predict whether a given cortical field is behaviourally affected (Supplementary Table 3).

### Calcium accumulation is non-topographic and encompasses low myelin borders

The pathology maps (Fig. 2) provide a novel overview of calcium dysregulation in early-stage ALS, which suggests that calcium accumulation is atopographic. We show significantly decreased nQSM in the low-myelin borders (between the UL and F fields) compared to the cortical fields themselves (averaged UL and F fields), specifically in Ls [borders: mean = −0.0116, SD = 0.002; cortical fields: mean = 0.0159, SD = 0.027; *t*(6) = −2.77; *P* = 0.033, *d* = −1.25, 95% CI (−2.68 0.12)] and L5a [borders: mean = −0.0078, SD = 0.002; cortical fields: M = 0.0046, SD = 0.010; *t*(6) = −3.09; *P* = 0.021, *d* = −1.50, 95% CI (−3.11 0.25)], but not in L5b [borders: mean = −0.0062, SD = 0.003; cortical fields: M = −0.0015, SD = 0.018; *t*(6) = −0.77; *P* = 0.468, *d* = −0.30, 95% CI (−1.41 0.72)] or L6 [borders: mean = −0.0074, SD = 0.004; cortical fields: mean = −0.0021, SD = 0.015; *t*(6) = −1.30; *P* = 0.242, *d* = −0.42, 95% CI (−1.40 0.45)].

### Multiple hot spots of pathology in M1

We also addressed whether *in vivo* pathology maps favour the hypothesis of one large pathology hot spot or multiple small hot spots of pathology. The maps reveal that pathology occurs in multiple, small areas, often beyond the first-affected cortical field (Fig. 2). For example, Patient P1 shows iron accumulation in an area between the left LL and UL fields compared to the control, while this patient shows demyelination in the (first-affected) left UL field.

### Calcium accumulation precedes demyelination

**Figure 4:**
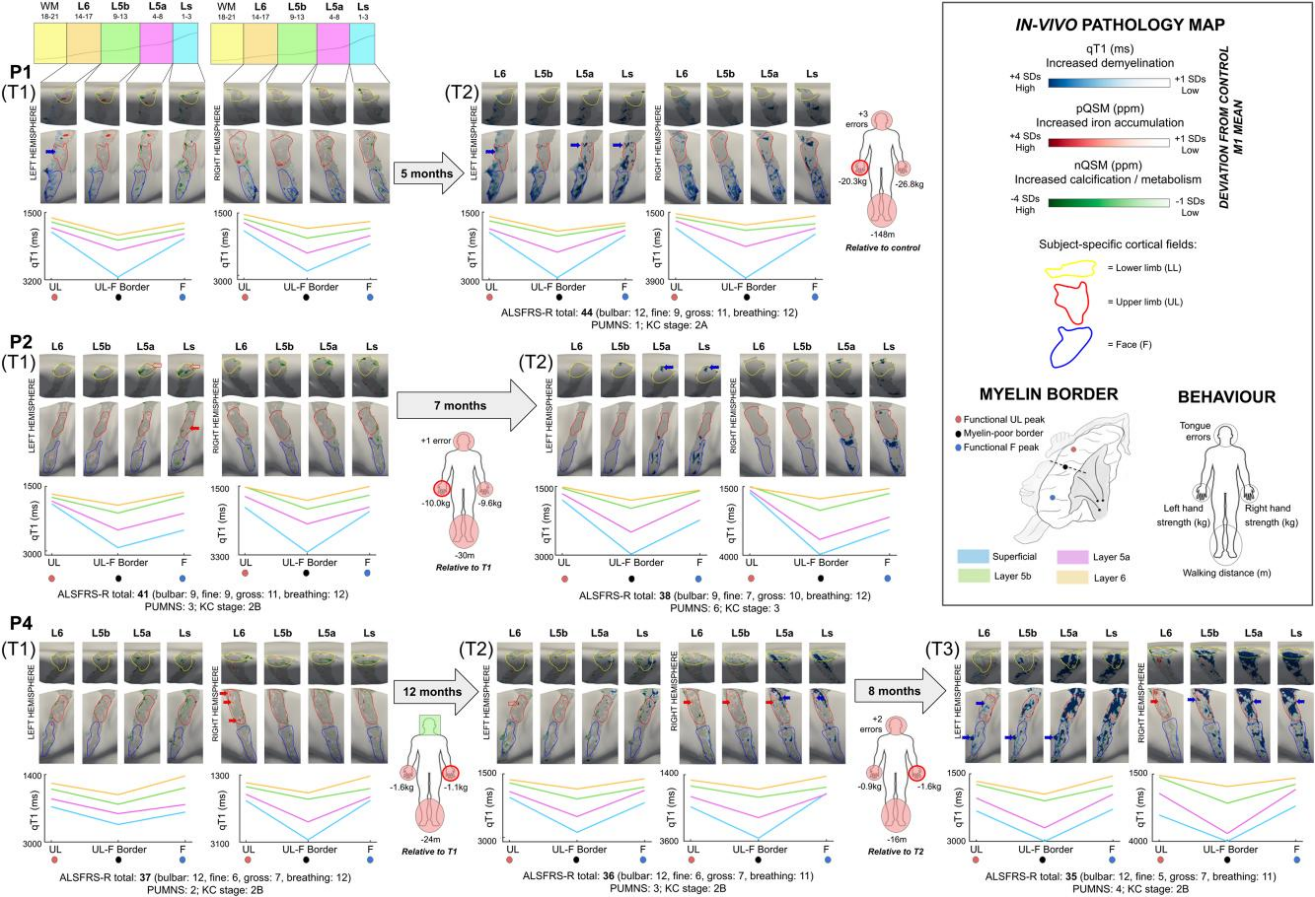
**Longitudinal multimodal *in vivo* pathology maps in ALS patients.** Individualized *in vivo* pathology maps were generated by thresholding the displayed value ranges at each layer to show increased qT1 and pQSM (+1 to +4 SD), and reduced nQSM (−1 to −4 SD), with respect to the mean M1 value of the matched control at baseline. Row 1 shows the pathology maps for Patient P1 at T1 (time point 1) and T2 (time point 2), row 2 shows the pathology maps for Patient P2 at T1 and T2 and row 3 shows the pathology maps for Patient P4 at T1, T2 and T3 (time point 3). Pathology maps: red and blue arrows on the pathology maps indicate iron accumulation and demyelination in the cortical field corresponding to symptom onset site, respectively. Body maps: red-outlined circles on the body maps indicate the onset site of the patient, while filled red and green circles indicate impaired or better motor function in the circled body part compared to the matched control, respectively. QSM data were excluded at T2 for Patients P1 and P2 due to severe artefacts. Clinical information: ALS Functional Rating Scale—Revised (ALSFRS-R)^31^ indicates disease severity, where lower values indicate greater impairment, with subscores for fine, gross and bulbar motor function. The Penn Upper Motor Neuron Scale (PUMNS)^29^ score indicates clinical signs of upper motor neuron involvement, with higher scores indicating greater impairment. The King’s College (KC) stage^32^ indicates disease progression based on ALSFRS-R score^33^: stage 2A reflects the involvement of one body part and that a clinical diagnosis has taken place, while stage 2B and stage 3 reflect the subsequent involvement of second and third body parts, respectively. L6 = layer 6; L5b = layer 5b; L5a = layer 5a; Ls = superficial layer; nQSM = negative QSM; pQSM = positive QSM; qT1 = quantitative T1; LL = lower limb; UL = upper limb; F = face.

Out of the 12 patients, three (Patients P1, P2 and P4) were also measured longitudinally (Fig. 4). To provide an overview over disease progression, we quantified the increase in the percentage of demyelinated vertices (+2 SD compared to matched control mean) in the first-affected cortical field between timepoints (Supplementary Table 4). In addition, we show that the topography of pathological calcium (vertices with QSM values > than −2 SD from the matched control mean) at T1 predicts myelination at T2 and T3, most strongly in Ls (Supplementary Table 5). We also show that the topography of pathological iron (vertices with QSM values > +2 SD from the matched control M1 mean) at T1 predicts myelination at T2 (but not T3), specifically in L6 (Supplementary Table 6). However, the latter effect did not survive correction for multiple comparisons.

### Disrupted low myelin borders with disease progression

We also addressed whether the low-myelin borders that separate the F and UL cortical fields in M1 are affected or preserved in ALS, to identify their role in disease progression. The longitudinal pathology maps show demyelination of the F-UL border at T2 compared to T1 (Patients P1, P2 and P4 in Fig. 4), and at T3 compared to T2 (Patient P4 in Fig. 4). Based on visual inspection and the quantified demyelination (Supplementary Table 4), this effect is largely restricted to Ls and L5a, where the low myelin borders are typically most defined in M1 of healthy adults.^10,23^

### Multimodal MRI markers of slow disease progression

To identify cortical markers related to slow disease progression, we compared the individualized *in vivo* pathology maps between the very slow progressing patient (Patient P4) and all other patients. We show that Patient P4 shows a significantly higher percentage of abnormal pQSM vertices (i.e. increased iron accumulation) in L5b [*t*(6) = 17.07, *P* = 1 × 10^−5^, *d* = 6.45, 95% CI (2.84 10.08)] and L5a [*t*(6) = 5.53, *P* = 0.001, *d* = 2.09, 95% CI (.70 3.43)], in addition to a significantly higher percentage of abnormal nQSM vertices (i.e. increased calcium accumulation) in L5a [*t*(6) = 4.96, *P* = 0.003, *d* = 1.88, 95% CI (.58 3.13)] at T1, compared to all other patients. In addition, our data show that Patient P4 shows a significantly lower percentage of abnormal qT1 vertices (i.e. less demyelination) in all layers compared to the other patients at T1 [L6: *t*(10) = −2.40, *P* = 0.037, *d* = −0.73, 95% CI (−1.38 −0.04); L5b: *t*(10) = −2.79, *P* = 0.019, *d* = −0.75, 95% CI (−1.41 −0.06); L5a: *t*(10) = −2.33, *P* = 0.042, *d* = −0.70, 95% CI (−1.35 −0.02); Ls: *t*(10) = −2.40, *P* = 0.038, *d* = −0.72, 95% CI (−1.38 −0.04)].

## Discussion

**Figure 5:**
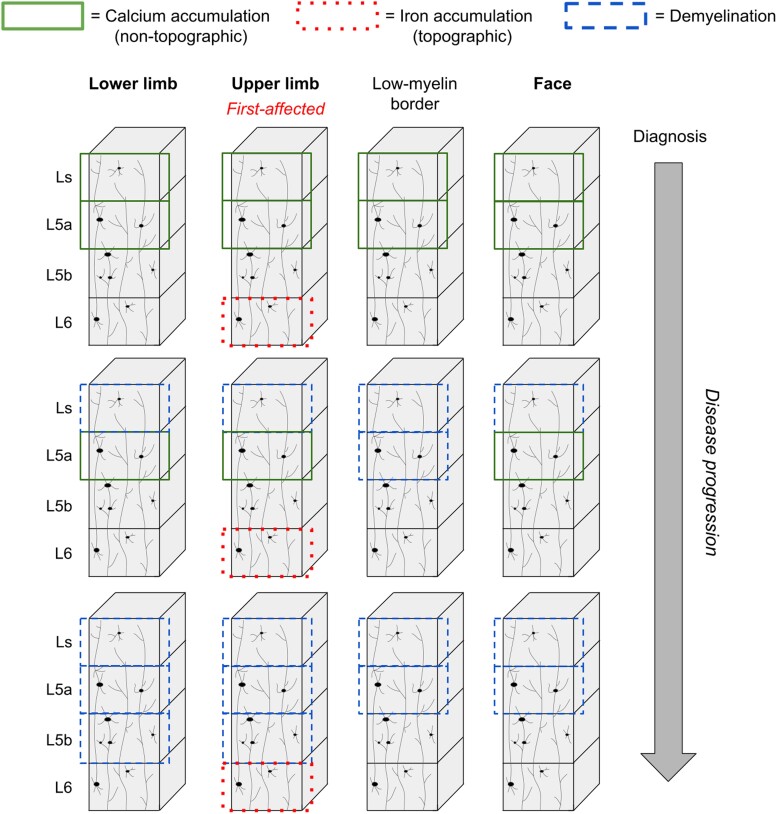
**Model of *in vivo* M1 pathology progression in ALS.** Schematic depiction of the layer-specific pathology features shown in ALS patients compared to matched healthy controls in the present study. In the early pathology stage, we demonstrate topographic (i.e. most in first-affected cortical field—example upper limb shown here) iron accumulation in L6 and non-topographic (i.e. more widespread) calcium accumulation in Ls and L5a (see Table 3 and Figs 2 and 3). With disease progression, we highlight increasing demyelination (Supplementary Table 4), particularly in Ls and L5a and in the low-myelin borders between adjacent cortical fields (see Fig. 4, based on visual inspection), corresponding to calcium accumulation at earlier time points (Supplementary Table 5). L6 = layer 6; L5b = layer 5b; L5a = layer 5a; Ls = superficial layer.

ALS is a rapidly progressing neurodegenerative disease characterized by the loss of motor control.^1^ The present study aimed to systematically describe the *in vivo* pathology of M1 in early stage ALS patients. Given the layer-specific and topographic signature of pathology in ALS,^5^ we combined submillimetre structural 7 T MRI data (qT1, QSM) and automated layer modelling with cortical field-specific analyses to calculate precise *in vivo* histology profiles of the patients. Our data reveal critical insights into the *in vivo* pathology of ALS that can be summarized as (i) preserved mean cortical thickness and preserved layer architecture of M1; (ii) layer-specific iron (layer 6) and calcium (layer 5a, superficial layer) accumulation; (iii) topographic iron accumulation, but atopographic calcium accumulation; (iv) disrupted low myelin borders with increased calcium; (v) later demyelination may occur more in the earlier hot spots of calcium accumulation and does therefore not show a strictly topographic profile; (vi) demyelination characterizes disease progression (particularly in layer 5a and superficial layer); and (vii) the very slow-progressing patient has a distinct pathology profile in M1 compared to the other patients. Overall, our study provides novel insights into the *in vivo* M1 pathology in ALS, offering new perspectives into disease mechanisms and diagnosis (Fig. 5).

Across cortical depth in M1, we show no significant differences in microstructure or mean cortical thickness between patients and controls. This contrasts previous evidence of reduced cortical thickness in ALS patients compared to healthy controls at varying magnetic resonance field strengths.^16,56-58^ However, the patients in the present study show a relatively slower disease progression rate compared to a previous 7 T study.^16^ Moreover, our cortical thickness estimates of healthy controls are comparable to previous automated 7 T estimates.^16,59^ We show a trend towards reduced mean cortical thickness in the F field of patients compared to controls. This field-specific trend may reflect a higher vulnerability of this field to neurodegeneration, as it has been shown that myelination is reduced in the F field compared to the other cortical fields of M1.^10^ Our findings therefore confirm that layer-specific microstructural pathology in ALS is detectable in the absence of significant cortical atrophy.

Layer-specific analyses reveal that iron accumulation in ALS is specific to L6 of M1. Although this effect was moderate-to-large, it is critical to replicate this finding in a larger cohort of patients given the high heterogeneity of the disorder. Nevertheless, this finding extends previous studies showing deep iron accumulation in ALS, which is considered to reflect neuroinflammation and activated microglia.^6,15^ This suggests that iron accumulation in L6 may provide an earlier marker of pathology than the well established degeneration of L5(b).^5^ Our data also show that iron accumulates most strongly in the first-affected cortical field, as expected,^17-19^ and that this is specific to L6. However, in our data, the degree of iron accumulation (% of pQSM pathology) does not predict whether a given cortical field is behaviourally affected. This may suggest that iron accumulation largely serves as a specific marker for the first-affected cortical field, which may support early diagnosis, and that this marker is a consequence rather than the cause of the disease.

Surface mapping reveals multiple, small hot spots of pathology rather than one large hot spot. For example, one patient (Patient P1) shows L6 iron accumulation in an area between the LL and UL fields, which may reflect pathology in the torso region where impairment is difficult to detect. Another patient (Patient P2) shows iron accumulation in Ls-L5a of the first-affected (UL) field, deviating from the expected location in L6, but in all layers of the contralateral F field. The latter may reflect the quantitatively impaired tongue function in this patient, highlighting challenges in detecting bulbar symptoms clinically.^54^ This demonstrates potential mismatches between brain and behaviour in ALS,^60^ emphasizing the utility of MRI for individualized medicine.

We also investigated calcium dysregulation, demonstrating that Ls (including L2-L3) and L5a show calcium accumulation in ALS. In addition, we show that calcium accumulation is higher in the areas between cortical fields, where the low myelin borders are located, compared to the cortical fields themselves. This highlights the atopographic profile of calcium accumulation in ALS, in contrast to the more topographic iron accumulation. Previous evidence has shown disrupted calcium homeostasis in ALS,^7^ where increased intracellular calcium is associated with glutamate excitotoxicity in animal models.^20,21^ Increased calcium in humans may reflect similar mechanisms, including the increased activity of M1 in ALS^61^ or calcifications in blood vessels related to poor brain health.^62^ Interestingly, patients with pronounced calcium accumulation (Patients P8 and P12) largely show more focal behavioural impairment in our data. This may suggest that calcium accumulation provides an early disease marker, which may be related to compensation, as has been suggested for increased activity in M1.^61^

Based on a small sample of patients with longitudinal data, we provide evidence towards widespread demyelination of M1 with disease progression. We also show that later demyelination is higher at previous calcium accumulation hot spots, whereas the relation to previous iron accumulation hot spots is weaker. Moreover, the low myelin borders between cortical fields that are disrupted with disease progression show earlier increased calcium accumulation compared to cortical fields. Overall, this points towards a partly atopographic character of disease spread in ALS, and a potential role of the low myelin borders, which are connected to multiple body parts,^23,25^ in disease spread. These findings also suggest that metabolism disruption and inflammation may precede cell loss in ALS.^63^ An alternative perspective to this interpretation is that connections between low myelin borders and subcortical structures^25^ are more affected by pathology, compared to connections between cortical fields and subcortical structures. From this perspective, the affected border areas would not reflect markers of disease spread but would be the consequence of affected subcortical systems. Further investigation is needed to characterize the role of these atopographic disease mechanisms in ALS.

Finally, we also investigated whether the *in vivo* pathology profile of a very slow progressing patient (Patient P4) is distinct from the other patients. We demonstrate that Patient P4 shows increased iron accumulation in L5b and L5a, and increased calcium accumulation in L5a, compared to other patients. Moreover, Patient P4 shows decreased demyelination in all layers compared to the other patients. These results suggest that Patient P4, despite the long disease duration, may be in the earlier stage of cortical pathology (Fig. 5) that is characterized by increased substance accumulation. One may interpret this finding to argue that there are distinct pathology profiles for disease duration (i.e. longer survival associated with substance accumulation) and disease progression (i.e. increased disease severity associated with demyelination). More specifically, one may argue that the ‘turning point’ between substance accumulation and cell loss is delayed in the slow-progressing patient, rather than a delay in substance accumulation as such. These assumptions must be verified in a larger patient sample with more slow- and more fast-progressing patients to avoid overinterpretation based on single patient profiles.

With respect to the applied methodology, although the method we used to define cortical layers in M1 is based on an *in vivo–ex vivo* validation model,^9,10^ we cannot guarantee that our layer definitions correspond to the exact biological layers. This limitation should be addressed with more extensive *in vivo–ex vivo* validation studies. Finally, although quantitative and validated markers of cortical microstructure were used here, we cannot be certain of the exact tissue properties measured.

In summary, we provide novel insights into the *in vivo* pathology of ALS patients using topographic layer imaging. We show that layer-specific changes in tissue microstructure precede gross cortical atrophy. We highlight the topographic nature of iron accumulation as a marker for diagnosis, while atopographic calcium accumulation may precede demyelination and cell loss. The role of the low myelin borders between cortical fields, which show particularly high calcium accumulation, in disease progression needs further clarification. Finally, we highlight the distinct pathology profile of a very slow progressing patient, where increased substance accumulation and reduced demyelination may indicate a lack of pathology progression despite a long disease duration. Our efficient 7 T MRI scanning protocol, and use of open-source analysis tools, make our approach accessible to large cohort investigations.

## Supplementary Material

awad351_Supplementary_Data

## Data Availability

The data used in the present study have been made available in a public repository (https://github.com/alicianorthall/In vivo-Pathology-ALS).

## References

[1] Al-Chalabi A , Hardiman O, Kiernan MC, Chiò A, Rix-Brooks B, van den Berg LH. Amyotrophic lateral sclerosis: Moving towards a new classification system. Lancet Neurol. 2016;15:1182–1194. 27647646 10.1016/S1474-4422(16)30199-5

[2] Norris F , Shepherd R, Denys E, et al Onset, natural history and outcome in idiopathic adult motor neuron disease. J Neurol Sci. 1993;118:48–55. 8229050 10.1016/0022-510x(93)90245-t

[3] Ravits JM , La Spada AR. ALS Motor phenotype heterogeneity, focality, and spread: Deconstructing motor neuron degeneration. Neurology. 2009;73:805–811. 19738176 10.1212/WNL.0b013e3181b6bbbd PMC2739608

[4] Chio A , Logroscino G, Hardiman O, et al Prognostic factors in ALS: A critical review. Amyotroph Lateral Scler. 2009;10:310–323. 19922118 10.3109/17482960802566824 PMC3515205

[5] Hammer RP , Tomiyasu U, Scheibel AB. Degeneration of the human Betz cell due to amyotrophic lateral sclerosis. Exp Neurol. 1979;63:336–346. 437007 10.1016/0014-4886(79)90129-8

[6] Kwan JY , Jeong SY, Van Gelderen P, et al Iron accumulation in deep cortical layers accounts for MRI signal abnormalities in ALS: Correlating 7 Tesla MRI and pathology. PLoS One. 2012;7:e35241. 22529995 10.1371/journal.pone.0035241 PMC3328441

[7] Appel SH , Beers D, Siklos L, Engelhardt JI, Mosier DR. Calcium: The darth vader of ALS. Amyotroph Lateral Scler Other Motor Neuron Disord. 2001;2:47–54. 11465925

[8] Sekiguchi T , Kanouchi T, Shibuya K, et al Spreading of amyotrophic lateral sclerosis lesions--multifocal hits and local propagation? J Neurol Neurosurg Psychiatry. 2014;85:85–91. 24027298 10.1136/jnnp-2013-305617

[9] Huber L , Handwerker DA, Jangraw DC, et al High-resolution CBV-fMRI allows mapping of laminar activity and connectivity of cortical input and output in human M1. Neuron. 2017;96:1253–1263.e7. 29224727 10.1016/j.neuron.2017.11.005 PMC5739950

[10] Northall A , Doehler J, Weber M, Vielhaber S, Schreiber S, Kuehn E. Layer-specific vulnerability is a mechanism of topographic map aging. Neurobiol Aging. 2023;128:17–32. 37141729 10.1016/j.neurobiolaging.2023.04.002

[11] Doehler J , Northall A, Liu P, et al The 3D structural architecture of the human hand area is nontopographic. J Neurosci. 2023;43:3456–3476. 37001994 10.1523/JNEUROSCI.1692-22.2023 PMC10184749

[12] Acosta-Cabronero J , Machts J, Schreiber S, et al Quantitative susceptibility MRI to detect brain iron in amyotrophic lateral sclerosis. Radiology. 2018;289:195–203. 30040038 10.1148/radiol.2018180112 PMC6166868

[13] Adachi Y , Sato N, Saito Y, et al Usefulness of SWI for the detection of iron in the motor cortex in amyotrophic lateral sclerosis. J Neuroimaging. 2015;25:443–451. 24888543 10.1111/jon.12127

[14] Wang C , Foxley S, Ansorge O, et al Methods for quantitative susceptibility and R2* mapping in whole post-mortem brains at 7T applied to amyotrophic lateral sclerosis. NeuroImage. 2020;222:117216. 32745677 10.1016/j.neuroimage.2020.117216 PMC7775972

[15] Pallebage-Gamarallage M , Foxley S, Menke RAL, et al Dissecting the pathobiology of altered MRI signal in amyotrophic lateral sclerosis: A post mortem whole brain sampling strategy for the integration of ultra-high-field MRI and quantitative neuropathology. BMC Neurosci. 2018;19:1–24. 29529995 10.1186/s12868-018-0416-1 PMC5848544

[16] Cosottini M , Donatelli G, Costagli M, et al High-Resolution 7T MR imaging of the motor Cortex in amyotrophic lateral sclerosis. Am J Neuroradiol. 2016;37:455–461. 26680464 10.3174/ajnr.A4562 PMC7960124

[17] Costagli M , Donatelli G, Biagi L, et al Magnetic susceptibility in the deep layers of the primary motor cortex in amyotrophic lateral sclerosis. NeuroImage Clin. 2016;12:965–969. 27995062 10.1016/j.nicl.2016.04.011 PMC5153607

[18] Donatelli G , Caldarazzo Ienco E, Costagli M, et al MRI Cortical feature of bulbar impairment in patients with amyotrophic lateral sclerosis. NeuroImage Clin. 2019;24:101934. 31377555 10.1016/j.nicl.2019.101934 PMC6698695

[19] Donatelli G , Costagli M, Cecchi P, et al Motor cortical patterns of upper motor neuron pathology in amyotrophic lateral sclerosis: A 3 T MRI study with iron-sensitive sequences. NeuroImage Clin. 2022;35:103138. 36002961 10.1016/j.nicl.2022.103138 PMC9421531

[20] Kruman II , Pedersen WA, Springer JE, Mattson MP. ALS-linked Cu/Zn–SOD mutation increases vulnerability of motor neurons to excitotoxicity by a mechanism involving increased oxidative stress and perturbed calcium homeostasis. Exp Neurol. 1999;160:28–39. 10630188 10.1006/exnr.1999.7190

[21] Siklós L , Engelhardt J, Harati Y, Smith RG, Joó F, Appel SH. Ultrastructural evidence for altered calcium in motor nerve terminals in amyotrophc lateral sclerosis. Ann Neurol. 1996;39:203–216. 8967752 10.1002/ana.410390210

[22] Eisen A , Pant B, Stewart H. Cortical excitability in amyotrophic lateral sclerosis: A clue to pathogenesis. Can J Neurol Sci. 1993;20:11–16. 8096792 10.1017/s031716710004734x

[23] Kuehn E , Dinse J, Jakobsen E, et al Body topography parcellates human sensory and motor cortex. Cereb Cortex. 2017;27:3790–3805. 28184419 10.1093/cercor/bhx026 PMC6248394

[24] Schreiber S , Northall A, Weber M, Vielhaber S, Kuehn E. Topographical layer imaging as a tool to track neurodegenerative disease spread in M1. Nat Rev Neurosci. 2021;22:68–69. 33154581 10.1038/s41583-020-00404-w

[25] Gordon EM , Chauvin RJ, Van AN, et al A somato-cognitive action network alternates with effector regions in motor cortex. Nature. 2023;617:351–359. 37076628 10.1038/s41586-023-05964-2 PMC10172144

[26] Bartzokis G , Lu PH, Mintz J. Human brain myelination and amyloid beta deposition in Alzheimer’s disease. Alzheimers Dement. 2007;3:122–125. 18596894 10.1016/j.jalz.2007.01.019 PMC2442864

[27] Stüber C , Morawski M, Schäfer A, et al Myelin and iron concentration in the human brain: A quantitative study of MRI contrast. Neuroimage. 2014;93:95–106. 24607447 10.1016/j.neuroimage.2014.02.026

[28] Wang Y , Spincemaille P, Liu Z, et al Clinical quantitative susceptibility mapping (QSM): Biometal imaging and its emerging roles in patient care. J Magn Reson Imaging. 2017;46:951–971. 28295954 10.1002/jmri.25693 PMC5592126

[29] Quinn C , Edmundson C, Dahodwala N, et al Reliable and efficient scale to assess upper motor neuron disease burden in amyotrophic lateral sclerosis. Muscle & Nerve. 2020;61:508–511. 31743477 10.1002/mus.26764

[30] Brooks BR , Miller RG, Swash M, Munsat TL. El escorial revisited: Revised criteria for the diagnosis of amyotrophic lateral sclerosis. Amyotroph Lateral Scler Other Motor Neuron Disord. 2000;1:293–299. 11464847 10.1080/146608200300079536

[31] Cedarbaum JM , Stambler N, Malta E, et al The ALSFRS-R: A revised ALS functional rating scale that incorporates assessments of respiratory function. J Neurol Sci. 1999;169:13–21. 10540002 10.1016/s0022-510x(99)00210-5

[32] Roche JC , Rojas-Garcia R, Scott KM, et al A proposed staging system for amyotrophic lateral sclerosis. Brain. 2012;135:847–852. 22271664 10.1093/brain/awr351 PMC3286327

[33] Balendra R , Jones A, Jivraj N, et al Estimating clinical stage of amyotrophic lateral sclerosis from the ALS functional rating scale. Amyotroph Lateral Scler Frontotemporal Degener. 2014;15:279–284. 24720420 10.3109/21678421.2014.897357

[34] Moore SR , Gresham LS, Bromberg MB, Kasarkis EJ, Smith RA. A self report measure of affective lability. J Neurol Neurosurg Psychiatry. 1997;63:89–93. 9221973 10.1136/jnnp.63.1.89 PMC2169647

[35] Smith RA , Macklin EA, Myers KJ, et al Assessment of bulbar function in amyotrophic lateral sclerosis: Validation of a selfreport scale (center for neurologic study bulbar function scale). Eur J Neurol. 2018;25:907–e66. 29577526 10.1111/ene.13638 PMC6005752

[36] Marques JP , Kober T, Krueger G, Van Der Zwaag W, Van De Moortele PF, Gruetter R. MP2RAGE, a self bias-field corrected sequence for improved segmentation and T1-mapping at high field. Neuroimage. 2010;49:1271–1281. 19819338 10.1016/j.neuroimage.2009.10.002

[37] Bazin PL , Weiss M, Dinse J, Schäfer A, Trampel R, Turner R. A computational framework for ultra-high resolution cortical segmentation at 7 tesla. NeuroImage. 2014;93:201–209. 23623972 10.1016/j.neuroimage.2013.03.077

[38] Mcauliffe MJ , Lalonde FM, Mcgarry D, Gandler W, Csaky K, Trus BL. Medical image processing, analysis and visualization in clinical research. In: Proceedings 14th IEEE Symposium on Computer-Based Medical Systems. IEEE Computer Society. 2001:381–386.

[39] Bazin PL , Pham DL. Homeomorphic brain image segmentation with topological and statistical atlases. Med Image Anal. 2008;12:616–625. 18640069 10.1016/j.media.2008.06.008 PMC2562468

[40] Han X , Pham DL, Tosun D, Rettmann ME, Xu C, Prince JL. CRUISE: Cortical reconstruction using implicit surface evolution. NeuroImage. 2004;23:997–1012. 15528100 10.1016/j.neuroimage.2004.06.043

[41] Waehnert MD , Dinse J, Weiss M, et al Anatomically motivated modeling of cortical laminae. Neuroimage. 2014;93:210–220. 23603284 10.1016/j.neuroimage.2013.03.078

[42] Waehnert MD , Dinse J, Schäfer A, et al A subject-specific framework for in vivo myeloarchitectonic analysis using high resolution quantitative MRI. Neuroimage. 2016;125:94–107. 26455795 10.1016/j.neuroimage.2015.10.001

[43] Acosta-Cabronero J , Milovic C, Mattern H, Tejos C, Speck O, Callaghan MF. A robust multi-scale approach to quantitative susceptibility mapping. Neuroimage. 2018;183:7–24. 30075277 10.1016/j.neuroimage.2018.07.065 PMC6215336

[44] Betts MJ , Acosta-Cabronero J, Cardenas-Blanco A, Nestor PJ, Düzel E. High-resolution characterisation of the aging brain using simultaneous quantitative susceptibility mapping (QSM) and R2* measurements at 7 T. Neuroimage. 2016;138:43–63. 27181761 10.1016/j.neuroimage.2016.05.024

[45] Sereno MI , Lutti A, Weiskopf N, Dick F. Mapping the human cortical surface by combining quantitative T1 with retinotopy. Cereb Cortex. 2013;23:2261–2268. 22826609 10.1093/cercor/bhs213 PMC3729202

[46] Dinse J , Härtwich N, Waehnert MD, et al A cytoarchitecturedriven myelin model reveals area-specific signatures in human primary and secondary areas using ultra-high resolution in vivo brain MRI. Neuroimage. 2015;114:71–87. 25896931 10.1016/j.neuroimage.2015.04.023

[47] Vogt C , Vogt O. Allgemeine ergebnisse unserer hirnforschung [General results of our brain research]. Zeitschrift für Augenheilkunde. 1919;25:273–462.

[48] Glasser MF , Coalson TS, Robinson EC, et al A multi-modal parcellation of human cerebral cortex. Nature. 2016;536:171–178. 27437579 10.1038/nature18933 PMC4990127

[49] Enright PL . The six-minute walk test. Respir Care. 2003;48:783–785. 12890299

[50] Peolsson A , Hedlund R, Öberg B. Intra-and inter-tester reliability and reference values for hand strength. J Rehabil Med. 2001;33:36–41. 11480468 10.1080/165019701300006524

[51] Tiffin J , Asher EJ. The purdue pegboard: Norms and studies of reliability and validity. J Appl Psychol. 1948;32:234–247. 18867059 10.1037/h0061266

[52] Matthews CG , Klove H. *Instruction manual for the Adult Neuropsychology Test Battery*. WI: University of Wisconsin Medical School; 1964:36.

[53] Fleishman EA . A modified administration procedure for the O’Connor finger dexterity test. J Appl Psychol. 1953;37:191–194.

[54] Northall A , Mukhopadhyay B, Weber M, et al An automated tongue tracker for quantifying bulbar function in ALS. Front Neurol. 2022;13:838191. 35280269 10.3389/fneur.2022.838191 PMC8914067

[55] Sawilowsky SS . New effect size rules of thumb. J Mod Appl Stat Methods. 2009;8:597–599.

[56] Agosta F , Valsasina P, Riva N. The cortical signature of amyotrophic lateral sclerosis. PLoS One. 2012;7:e42816. 22880116 10.1371/journal.pone.0042816 PMC3412820

[57] Roccatagliata L , Bonzano L, Mancardi G, Canepa C, Caponnetto C. Detection of motor cortex thinning and corticospinal tract involvement by quantitative MRI in amyotrophic lateral sclerosis. Amyotroph Lateral Scler. 2009;10:47–52. 18622772 10.1080/17482960802267530

[58] Donatelli G , Retico A, Caldarazzo Ienco E, et al Semiautomated evaluation of the primary motor Cortex in patients with amyotrophic lateral sclerosis at 3T. Am J Neuroradiol. 2018;39:63–69. 29122765 10.3174/ajnr.A5423 PMC7410679

[59] Lüsebrink F , Wollrab A, Speck O. Cortical thickness determination of the human brain using high resolution 3T and 7T MRI data. Neuroimage. 2013;70:122–131. 23261638 10.1016/j.neuroimage.2012.12.016

[60] Verstraete E , Turner MR, Grosskreutz J, Filippi M, Benatar M; attendees of the 4th NiSALS meeting. Mind the gap: the mismatch between clinical and imaging metrics in ALS. Amyotroph Lateral Scler Frontotemporal Degener. 2015;16:524–529. 26402254 10.3109/21678421.2015.1051989

[61] Proudfoot M , Bede P, Turner MR. Imaging cerebral activity in amyotrophic lateral sclerosis. Front Neurol. 2019;9:1148. 30671016 10.3389/fneur.2018.01148 PMC6332509

[62] Schreiber S , Bernal J, Arndt P, et al Brain vascular health in ALS is mediated through motor cortex microvascular integrity. Cells. 2023;12:957. 36980297 10.3390/cells12060957 PMC10047140

[63] Toft MH , Gredal O, Pakkenberg B. The size distribution of neurons in the motor cortex in amyotrophic lateral sclerosis. J Anat. 2005;207:399–407. 16191168 10.1111/j.1469-7580.2005.00465.x PMC1571546

